# “Hooking method” for hepatic inflow control: a new approach for laparoscopic Pringle maneuver

**DOI:** 10.1186/s12957-023-03149-9

**Published:** 2023-08-22

**Authors:** Yi Zhou, Yifan Wang, Jinliang Ma, Chuanhai Zhang

**Affiliations:** 1 Department of Hepatic Surgery, The First Affiliated Hospital of USTC, Division of Life Sciences and Medicine, University of Science and Technology of China, Hefei, Anhui 230001 China; 2 Department of Emergency Medicine, The First Affiliated Hospital of USTC, Division of Life Sciences and Medicine, University of Science and Technology of China, Hefei, Anhui 230001 China

**Keywords:** Laparoscopy hepatectomy, Pringle maneuver, Hepatic inflow control

## Abstract

**Background:**

The laparoscopic Pringle maneuver is crucial for controlling bleeding during laparoscopic hepatectomy. In this study, we introduce a new laparoscopic Pringle maneuver and preliminarily investigate its application in laparoscopic hepatectomy.

**Methods:**

We collected and analyzed the clinical data of 17 consecutive patients who underwent laparoscopic hepatectomy at the Department of Hepatic Surgery, the First Affiliated Hospital of the University of Science and Technology of China, from January 2022 to January 2023. All patients underwent the hooking method for intermittent occlusion of hepatic inflow. Intraoperative and postoperative clinical indices were observed and recorded.

**Results:**

All 17 patients underwent laparoscopic hepatectomy with hepatic inflow control using the hooking method. Four patients with adhesions under the hepatoduodenal ligament successfully had occlusion loops placed using the hooking method combined with Zhang’s modified method during surgery. The median occlusion time for the 17 patients was 34 (12–60) min, and the mean operation time was 210 ± 70 min. The mean intraoperative blood loss was 145 ± 86 ml, and no patients required intraoperative blood transfusion. The patients’ postoperative peak AST was 336 ± 183 U/L, and the postoperative peak ALT was 289 ± 159 U/L. Postoperative complications occurred in 2 patients (11.8%), including 1 Clavien-Dindo grade I and 1 Clavien-Dindo grade II complication. No Clavien-Dindo grade IIIa or higher complications or deaths occurred in any patient. None of the patients developed portal vein thrombosis or hepatic artery aneurysm formation. The median postoperative hospital stay was 6 (4–14) days.

**Conclusion:**

The hooking method combines the advantages of both intracorporeal Pringle maneuver and extracorporeal Pringle maneuver. It is a simple, safe, and effective method for controlling hepatic inflow and represents a promising approach for performing totally intracorporeal laparoscopic Pringle maneuver.

**Supplementary Information:**

The online version contains supplementary material available at 10.1186/s12957-023-03149-9.

## Introduction

As surgeons have enhanced their surgical skills, laparoscopic techniques can now be applied to all types of hepatectomy [1, 2]. Over the past few years, the amount of bleeding in laparoscopic hepatectomy has also decreased [3]. Furthermore, numerous advanced techniques and effective instruments can help reduce bleeding during liver surgery [4–6]. However, major laparoscopic hepatectomy still experiences more intraoperative bleeding than minor laparoscopic hepatectomy [7, 8]. The Pringle maneuver (PM) remains an essential method for controlling intraoperative bleeding in hepatectomy [9]. In open hepatectomy, PM can be performed safely, effectively, and easily with a cloth strip or directly with a vascular clamp. However, in laparoscopic hepatectomy, performing PM is not as straightforward.

In this paper, we introduce a fully intracorporeal laparoscopic PM; preliminarily assess its safety, efficacy, and simplicity; and compare the advantages and disadvantages of this method with other laparoscopic PM approaches.

## Materials and methods

From January 2022 to January 2023, a total of 17 consecutive patients underwent laparoscopic hepatectomy using the hooking method for hepatic inflow control at the Department of Hepatic Surgery, the First Affiliated Hospital of the University of Science and Technology of China. The procedures were performed by the same team, who had expertise in laparoscopic hepatectomy. Total laparoscopic hepatectomy was performed in all patients, while patients requiring conversion to laparotomy and those requiring concomitant surgery were excluded. All patients provided informed consent in accordance with the hospital ethics committee’s approval, which was in line with the provisions of the Declaration of Helsinki on Medical Ethics.

### Preparation before operation

**Fig. 1:**
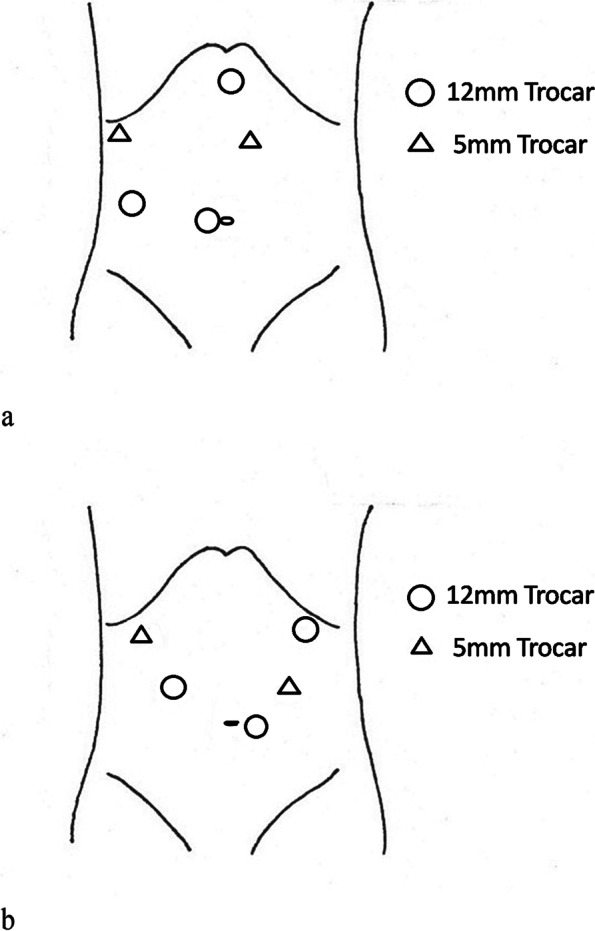
**a** Trocar’s location during resection of the left liver tumor. **b** Trocar’s location during resection of the right liver tumor

The patient was placed in a supine position, and the “five-hole” approach for trocar placement (Fig. 1a, b) consisted of two 5-mm and three additional 12-mm trocars. Carbon dioxide pneumoperitoneum was established at a pressure of 12–15 mmHg. The central venous pressure was maintained below 5-mm H_2_O. After opening the lesser omental sac using a harmonic scalpel (Harmonic, Ethicon), the procedure was initiated.

### Preparation of occlusion loop by “hooking method”

**Fig. 2:**
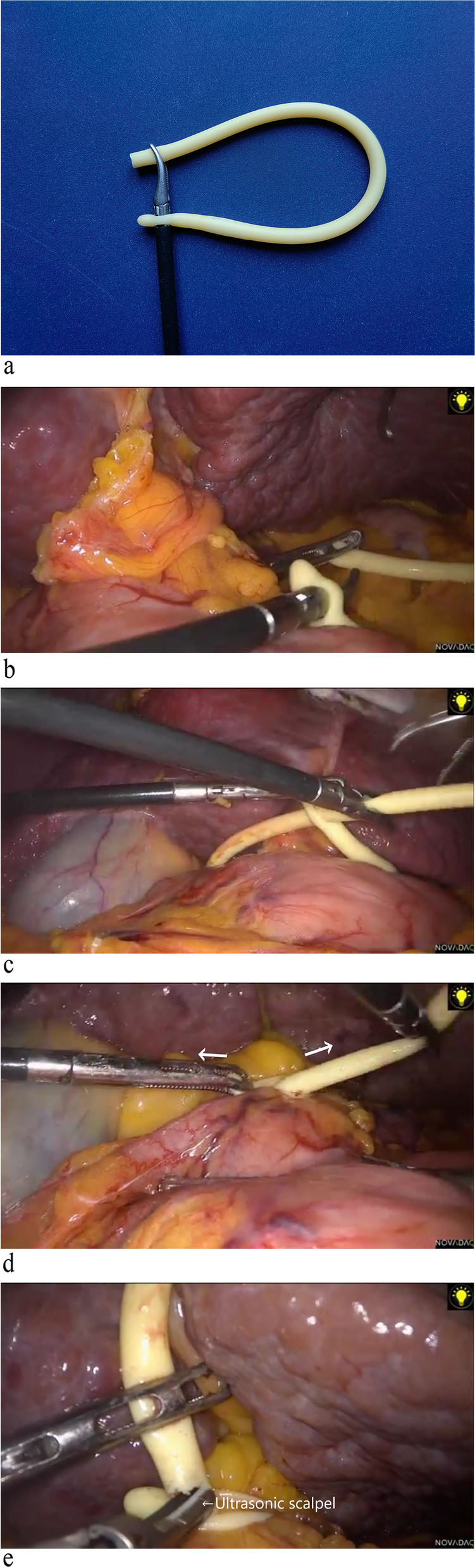
**a** Use the dissecting forceps to pass through the small hole in the front end of the catheter and clamp the tail of the catheter. **b** The operator’s left-hand grasper guides the catheter through the Winslow hole. **c** Form a loop around the hepatoduodenal ligament. **d** Tighten the loop with both hands in the direction of the arrow. **e** Cut about 1/2 or 1/3 of the circumference of the catheter body at the junction of the catheter loop and the catheter body with an ultrasonic scalpel

The extracorporeal fabrication process of the occlusion loop was the same as in the previously proposed Zhang’s modified method [10]. First, the tail of a 12-Fr or 14-Fr Foley catheter (hereinafter referred to as catheter) was cut off, leaving approximately 15 cm of the head. Outside the body, the surgeon held the tip of the dissecting forceps in their right hand and passed it through the small hole in the front end of the catheter (Fig. 2a). The catheter was then inserted into the abdominal cavity through the 12-mm trocar. The surgeon entered the 5-mm trocar below the right costal arch using grasping forceps with their left hand, passed through the Winslow foramen below the hepatoduodenal ligament, grasped the tail of the catheter, and guided the catheter through the Winslow foramen (Fig. 2b). At this point, the left-hand grasping forceps pulled down the catheter cover on the right-hand separating forceps, forming a loop around the hepatoduodenal ligament (Fig. 2c). The loop was then tightened (Fig. 2d), and about 1/2 of the circumference of the catheter body was cut at the junction between the loop and the catheter body with an ultrasonic scalpel (Fig. 2e). After opening a notch and loosening the loop, the fabrication of the occlusion loop was completed and set aside for later use (Video 1).

### Combined with Zhang’s modified method

In patients with adhesions below the hepatoduodenal ligament, adhesions should be separated as much as possible. Then, in combination with the previously proposed Zhang’s modified method [10], the surgeon can attempt to use the Goldenfinger to bluntly pass under the hepatoduodenal ligament. The tip of the Goldenfinger is then used to hook the silk thread prepared in advance at the tail end of the catheter, guiding the catheter through the ligament. Finally, the occlusion loop is created within the abdominal cavity using the method described above.

### Surgical procedure and application of “hooking method”

**Fig. 3:**
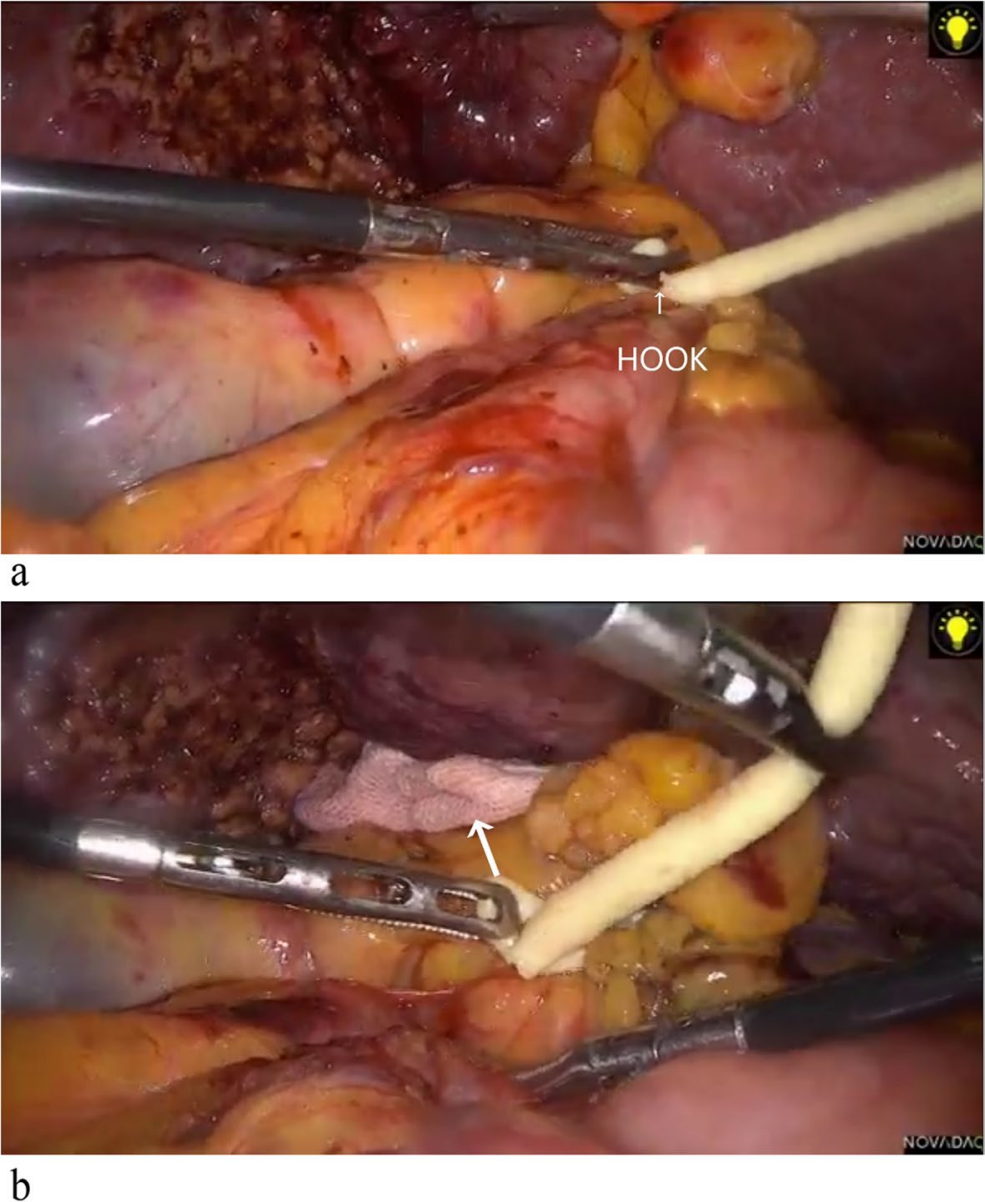
**a** Tighten the ring—hook the catheter head at the notch position. **b** Loosen the loop—hold the catheter head with a grasper and pull it up

**Fig. 4:**
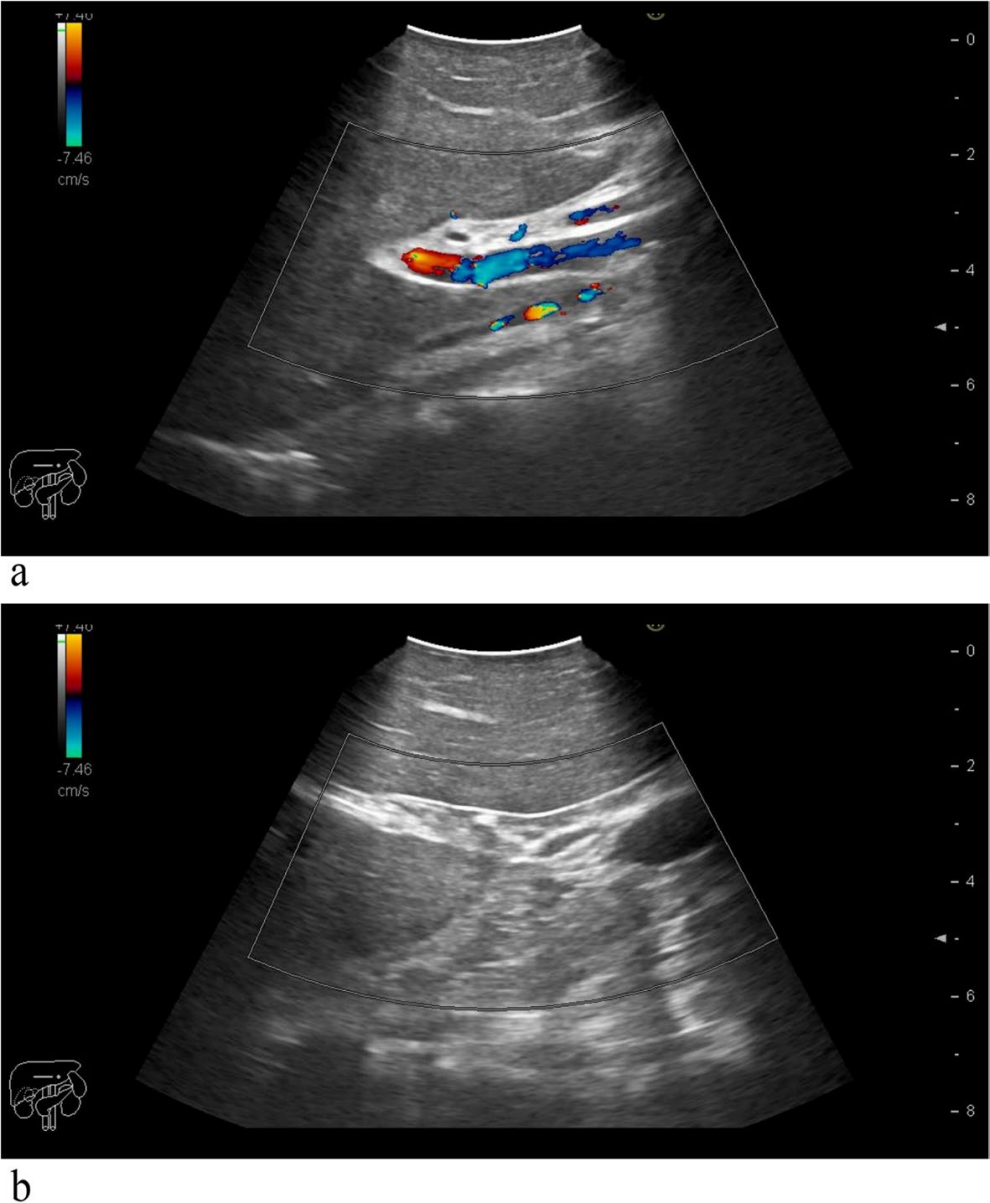
**a** Hepatic inflow intact before occlusion. **b** Hepatic inflow disappeared after occlusion

During the surgery, the harmonic scalpel was used for the “small steps and fast walking” approach to dissect the liver parenchyma, gradually resecting the target liver area. The ICG fluorescent staining technique (Stryker) and laparoscopic intraoperative ultrasound (Hitachi, Japan) were routinely employed for real-time guidance of the surgical procedure. When hepatic inflow occlusion was required during the operation, the catheter head could be hooked at the notch position after tightening the loop (Fig. 3a). To release the occlusion, the surgeon simply needed to clamp the head of the catheter with grasping forceps and lift upwards to release the catheter from the notch (Fig. 3b). Since the notch in the catheter could be hooked like a hook without falling off, we named this approach the “hooking method.” Laparoscopic ultrasound was used during the surgery to assess portal vein blood flow and determine whether the occlusion was complete (Fig. 4a, b). Each occlusion operation should not exceed 15 min, and the occlusion interval should not be less than 5 min. After completing the hepatectomy, suturing or electrocoagulation was used to treat bile leakage and hemorrhage of the liver section, depending on the situation. Abdominal drainage was placed as needed, and the specimen was removed. To conclude the procedure, the surgeon used the right-hand grasper to clamp the tip of the catheter, released the loop, and removed the occlusion loop from the 12-mm trocar.

### Observation indicators

We observed and recorded all patients’ intraoperative and postoperative clinical indicators. During the surgery, an anesthesiologist assessed and documented the duration of the hepatic portal occlusion, the number of occlusions, intraoperative blood loss, whether blood transfusion was needed, and the duration of the operation. Postoperatively, we recorded patients’ postoperative hospital stay, peak postoperative transaminase levels, and the occurrence of postoperative complications. Postoperative complications were graded according to the Clavien-Dindo classification method [11]. One week postoperatively, we conducted a CT examination on the patients to observe the presence of portal vein thrombosis or hepatic artery aneurysm formation. The clinical data of the patients were analyzed. Normally distributed data were expressed as mean ± standard deviation (SD); skewed data were expressed as median (range).

## Result

Among the 17 patients in this study, there were 14 males and 3 females, with an average age of 60 ± 10 years. All patients had no major underlying diseases, and their preoperative liver function was classified as Child–Pugh A. Six patients had cirrhosis. Regarding the tumor location, 5 patients had tumors in the left liver, 10 had tumors in the right liver, and 2 had one tumor in both the left and right liver (Table 1).

**Table 1:** Basic clinical characteristics and operation results of 17 patients

Case	Age	Sex	Liver cirrhosis	Tumor location	Surgical history	Surgical procedure	Occlusion time (min)	Blood loss (ml)	Operation time (min)	AST (U/L)	ALT (U/L)	Complications	POHS (days)	Pathology
1	73	M	Y	S2 + S7	LH	S2 + S7 NAH	13	200	312	521	493		11	HCC
2	44	M	Y	S6	-	S6 NAH	20	200	112	138	88		5	HCC
3	70	F	N	S2 + S3	LC	S2 + S3 AH	12	50	130	104	154		4	HCC
4	47	M	Y	S6	-	S6 NAH	23	200	220	170	128		6	HCC
5	53	M	Y	S5 + S6	-	S5 + S6 AH	35	200	250	298	256		5	HCC
6	65	M	N	S5	LH	S5 NAH	60	100	271	142	175		6	HCC
7	53	M	Y	S4	-	S4 AH	45	200	265	281	224	Pulmonary infection	14	HCC
8	55	F	Y	S6	-	S6 NAH	16	50	160	170	144		4	HCC
9	60	M	N	S6	-	S6 NAH	20	100	190	391	344		5	Hemangioma
10	60	M	Y	S5 + S8	-	S5 + S8 AH	60	200	323	722	682		6	HCC
11	57	M	Y	S4 + S6	LC	S4 AH + S6 NAH	60	300	248	652	406	Pleural effusion	5	MLC
12	53	M	Y	S2	-	S2 NAH	15	20	95	295	210		7	cHCC-CC
13	53	F	N	S2 + S3 + S4	-	S2 + S3 + S4 AH	14	300	295	378	310		5	Benign
14	55	M	Y	S3	-	S3 NAH	34	100	173	506	468		7	HCC
15	65	M	N	S8	-	S8 NAH	60	100	182	446	407		6	HCC
16	82	M	N	S4	-	S4 NAH	47	50	170	232	239		6	HCC
17	74	M	Y	S5	-	S5 NAH	40	100	170	271	181		10	HCC
Median (range)/mean ± SD^a^	60 ± 10						34 (12–60)	145 ± 86	210 ± 70	336 ± 183	289 ± 159		6 (4–14)	

*F* female, *M* male, *Y* yes, *N* no, *LH* laparoscopic hepatectomy, *LC* laparoscopic cholecystectomy, *NAH* non-anatomical hepatectomy, *AH* anatomical hepatectomy, *AST* aspartate transaminase, *ALT* alanine aminotransferase, *POHS* postoperative hospital stay, *HCC* hepatocellular carcinoma, *MLC* metastatic liver cancer, *cHCC-CC* combined hepatocellular cholangiocarcinoma

^a^Normally distributed data were expressed as mean ± standard deviation (SD); skewed data were expressed as median (range)

All patients successfully underwent laparoscopic hepatectomy using the hooking method to control hepatic inflow. Among them, there were 6 cases of laparoscopic anatomical liver resection and 11 cases of laparoscopic partial liver resection, with no conversions to open surgery. Four patients (23.5%) had a history of upper abdominal surgery, including 2 patients with a history of cholecystectomy and 2 patients with a history of laparoscopic liver resection. In these 4 patients, there were mild adhesions below the hepatoduodenal ligament, and the occlusion loop was successfully placed during the surgery in combination with Zhang’s modified method. The median hepatic pedicle occlusion time during surgery was 34 (12–60) min, and the average operation time was 210 ± 70 min. The average intraoperative blood loss was 145 ± 86 ml, and none of the patients required blood transfusion during the surgery.

In the 17 patients, the postoperative peak levels of AST were 336 ± 183 U/L, and the peak levels of ALT were 289 ± 159 U/L. Postoperatively, 2 patients (11.8%) experienced complications, with 1 case of Clavien-Dindo grade I and 1 case of Clavien-Dindo grade II complications. One patient developed pleural effusion, which resolved after conservative treatment, and another patient had a postoperative pulmonary infection that resolved after antibiotic treatment. No patients experienced Clavien-Dindo grade IIIa or higher complications or death. Furthermore, none of the patients developed portal vein thrombosis or hepatic artery aneurysm formation. The median postoperative hospital stay was 6 (4–14) days. Pathological results showed 13 patients had hepatocellular carcinoma, 1 patient had a mixed-type liver cancer, 1 patient had a benign lesion, 1 patient had hepatic hemangioma, and 1 patient had metastatic liver cancer (Table 1).

## Discussion

At present, numerous reports focus on hepatic inflow occlusion methods during laparoscopic hepatectomy, which can be divided into two main categories: intracorporeal Pringle maneuver (PM) and extracorporeal PM. Extracorporeal PM often involves using narrow tubing such as cloth strips or infusion tubes, passing them through thicker tubes like laparoscopic drainage tube, urinary catheter, tracheal catheter, or Tiemann catheter to form an occlusion loop [12–16]. The loop’s tail end is then passed through a trocar or an additional incision to facilitate occlusion and release operations. This method’s most apparent disadvantage is the need for an extra incision. Additionally, the occlusion loop extending from the exterior to the hepatic hilum may interfere with the surgeon’s view and the performance of laparoscopic instruments. If the external tube is not tightly clamped, it could easily cause pneumoperitoneum leakage. Moreover, reports suggest that narrow cloth strips may sometimes cause damage to the blood vessels within the hepatoduodenal ligament, leading to hepatic artery aneurysm and portal vein thrombosis [17, 18]. Another disadvantage of extracorporeal occlusion is that it can be challenging to perform when the patient is in the left lateral decubitus position [19].

Intracorporeal PM is performed entirely within the abdominal cavity. Unlike extracorporeal PM, the occlusion loop used in this method is completely placed inside the abdominal cavity. The loop can be made from a single rubber product such as a urinary catheter, the edge of a latex glove, or a T-tube [20–24]. Compared to extracorporeal PM, the difficulty of performing occlusion increases when done within the abdominal cavity using intracorporeal PM. During occlusion, the surgeon and assistant usually need to cooperate, pulling and maintaining tension while fixing the tail end with hemoclips to complete the procedure. To release the occlusion, specialized instruments are required to remove the hemoclips. This intricate occlusion process can potentially cause damage to surrounding tissues when there is significant bleeding in the abdominal cavity [15]. In emergency situations, removing hemoclips can be challenging [19]. Lastly, some believe that intracorporeal PM may not always achieve complete occlusion, and hemoclips can slip on the rubber tubing, further deteriorating the effectiveness of the occlusion [23] (Table 2).

**Table 2:** Details of the advantages of the various laparoscopic PM [12–17, 20–22, 24–26]

Year	Authors	Method	Advantages
2007	Maehara S, et al. [25]	Extracorporeal PM	Critical moments can be occluded safely and quickly No special tools like hemoclips needed, inexpensive Easy to release the occlusion
2009	Cho A, et al. [26]		
2011	Patriti A, et al. [27]		
2012	Rotellar F, et al. [15]		
2013	Okuda Y, et al. [12]		
2015	Mizuguchi T, et al. [16]		
2014	Dua MM, et al. [14]		
2019	Peng Y, et al. [13]		
2021	Onda S, [17]		
2012	Chao YJ, et al. [22]	Intracorporeal PM	No additional incision or trocar is required Does not obstruct the field of view or interfere with the operation Easy to perform in different positions
2018	Laurenzi A, et al. [23]		
2018	Huang JW, et al. [20]		
2020	Cai J, et al. [21]		

Therefore, we propose a new method for laparoscopic PM: the hooking method. Named for its resemblance to a hook gripping the front end of a urinary catheter, this method does not require additional hemoclips or specialized instruments, reducing extra costs and avoiding potential tissue damage from blindly clamping hemoclips. According to Huang et al. [20] study, the yellow color of the urinary catheter contrasts with the color of blood, making it more easily identifiable within the blood compared to materials like adhesive tape. Moreover, during instances of significant intra-abdominal bleeding that require rapid occlusion, the hooking method allows for the placement of a notch at an appropriate position on the occlusion loop beforehand. The surgeon can then perform the occlusion by grasping the head and tail of the urinary catheter using laparoscopic forceps and locking the catheter head into the pre-set notch. In emergency situations where quick release of the occlusion is needed, the surgeon can simply lift the catheter head, allowing it to disengage from the notch position. The entire occlusion and release process can be completed in a short time, and laparoscopic intraoperative ultrasound confirms the effectiveness of the hooking method, providing complete blockage of blood flow into the liver. This can help reduce intraoperative bleeding and decrease surgery time. Combined with intermittent blood flow occlusion, this method can also minimize ischemia–reperfusion injury to the liver [28]. In our study, the median hepatic portal occlusion time was 34 (12–60) min, with an average surgery duration of 210 ± 70 min. The average intraoperative blood loss was 145 ± 8 6 ml, with no patients requiring blood transfusion during surgery. Postoperative AST and ALT peak values were 336 ± 183 U/L and 289 ± 159 U/L, respectively.

Additionally, the hooking method as an intracorporeal PM technique retains the advantages of performing the procedure entirely through laparoscopy, without the need for additional incisions. It does not obstruct the surgeon’s visibility and is not limited by the patient’s position. Furthermore, the urinary catheter, a soft and elastic rubber material, is less likely to cause damage to the blood vessels within the hepatoduodenal ligament. In our study, no patients experienced hepatic artery aneurysms or portal vein thrombosis, demonstrating the safety and effectiveness of the hooking method.

Most of the current occluding devices mainly work by forming a freely contractible and releasable loop in the hepatoduodenal ligament region. Since the occlusion loop is usually soft, laparoscopic instruments are often required to guide the loop beneath the hepatoduodenal ligament. In our study, we still used a more conventional early method, selecting an appropriate trocar position and placing a 5-mm trocar on the right axillary line. Then, we used ordinary laparoscopic forceps to easily guide the placement of the occlusion loop through the Winslow foramen. Some studies have reported that using Biliary Scope, Endo Retract Maxi, Endo Retract mini, and 90° esophageal dissector can overcome trocar position limitations, but these methods require special instruments and may prolong the operation time [25, 26, 29]. In 2020, Liang et al. [21] proposed using forceps for gallstones as guidance, but this method, which utilizes open surgery instruments, may cause pneumoperitoneum leakage and subcutaneous emphysema during the operation. In 2018, Huang et al. [20] suggested that, under conditions of sufficient urinary catheter rigidity, there would be no need for fixed-position trocars or special instrument guidance; the catheter’s rigidity alone can easily pass through the Winslow foramen. However, we believe that since the urinary catheter is made of flexible material that bends easily, it is difficult to guide it when operating in the blind area beneath the hepatoduodenal ligament. When there is adhesion in the Winslow foramen, it is not easy to pass through with the catheter’s rigidity alone. Additionally, we inserted the dissecting forceps into the inherent side hole of the urinary catheter beforehand and then introduced the urinary catheter into the abdominal cavity. Huang et al.’s method subsequently requires the insertion of dissecting forceps into the inherent side hole of the catheter’s headend in the abdominal cavity, which is not an easy task to perform.

Previous studies have shown that it is difficult to place the occlusion loop in patients with a history of repeated hepatectomies [30]. However, we have proposed Zhang’s modified method [10], in which the surgeon sutures a thread to the tail of the urinary catheter beforehand. During the surgery, the surgeon uses a finger to create a tunnel beneath the hepatoduodenal ligament and hooks the thread at the tail of the urinary catheter with the fingertip to guide the catheter through the target hepatoduodenal area to form the occlusion loop. This method can be combined with the hooking method described in this article, that is, creating a hook-shaped notch after forming the occlusion loop. For patients with mild adhesions beneath the hepatoduodenal ligament, a blunt finger can be used to guide the placement of the occlusion loop through the Winslow foramen. In our study, two patients had a history of repeated hepatectomies, and there were mild adhesions beneath the hepatoduodenal ligament. Both patients successfully placed the occlusion loop using Zhang’s modified method.

There are still some deficiencies in this study: (1) Our study is a retrospective study with a small sample size and lacks a control group; (2) for patients with heavy adhesions below the hepatoduodenal ligament, it is difficult to place the occlusion loop through Winslow’s foramen. In such cases, the LSVC technique proposed by Onda et al. [17] in 2021 can be attempted, which directly uses vascular forceps to clamp the hepatoduodenal ligament. However, this technique may cause damage to the blood vessels within the hepatoduodenal ligament during clamping and requires additional incisions.

## Conclusions

The hooking method combines the advantages of intra- and extracorporeal PM and provides a safe, effective, and convenient way to control hepatic inflow in laparoscopic hepatectomy with accurate occlusion effect and simple operation. It has potential for clinical application.

### Supplementary Information

**Additional file 1:** **Video 1.**

## Data Availability

The datasets used or analyzed during the current study are available from the corresponding author on reasonable request.
